# Clinical Outcomes and Individualized Seed Implantation Planning for Iodine-125 Seeds Brachytherapy in Lymph Node Metastases

**DOI:** 10.7150/jca.126692

**Published:** 2026-01-30

**Authors:** Dongcun Huang, Zhihui Zhong, Fujun Zhang, Letao Lin

**Affiliations:** Department of Minimally Invasive Intervention, State Key Laboratory of Oncology in South China, Guangdong Key Laboratory of Nasopharyngeal Carcinoma Diagnosis and Therapy, Guangdong Provincial Clinical Research Center for Cancer, Sun Yat-sen University Cancer Center, Guangzhou 510060, P. R. China.

**Keywords:** brachytherapy, clinical outcomes, implantation planning, lymph node metastasis

## Abstract

**Background:**

Lymph node metastasis (LNM) critically influences cancer prognosis and treatment. This study explored the efficacy and prognostic factors of CT-guided radioactive iodine-125 (¹²⁵I) seed brachytherapy (RISB) for LNM and optimized the therapeutic dosage.

**Methods:**

We conducted a single-center retrospective cohort study analyzing 81 cases with histologically confirmed LNM (≤ 5 cm) from diverse primary cancers treated with CT-guided RISB. Postoperative dosimetric parameters (D90, D100, V90, V100, V150, V200) were assessed. Treatment response was evaluated at 6 months using RECIST 1.1, calculating the objective response rate (ORR) and local control rate (LCR). Patients were categorized into objective response and non-objective response groups based on treatment efficacy, and factors influencing treatment efficacy were identified through logistic regression analysis. Based on the ROC curve, the Youden index method was used to determine the dose optimization cutoff value.

**Results:**

The overall ORR was 71.6%, and LCR was 96.3%. The complication rate was 3.7%. Tumor size was an independent influencing factor for efficacy. Higher postoperative dosimetric parameters were associated with efficacy but were not independent influencing factors. ROC analysis identified the optimal D90 threshold as 102.7 Gy. The ORR in patients who achieved D90 > 102.7 Gy (n = 65, ORR = 81.54%) was significantly higher than in patients with D90 ≤ 102.7 Gy (n = 16, ORR = 31.25%) (p < 0.01). Complication rates did not differ between dose groups.

**Conclusion:**

Patients with LNM undergoing RISB can achieve a significantly higher ORR by ensuring a postoperative D90 > 102.7 Gy, without increasing the risk of complications. This dose threshold serves as a practical reference for clinical dose planning. Tumor size independently influences better response, guiding patient selection.

## Introduction

LNM is one of the critical prognostic factors in cancer, influencing therapeutic decisions and patient outcomes across multiple malignancies. The incidence of LNM exhibits substantial variability among different cancer types. In non-small cell lung cancer (NSCLC), LNM prevalence ranges from 25.0% to 38.2% 1, 2, while in thyroid cancer, central LNM occurs in 35.3% of cases, with micrometastases (≤ 0.28 cm) detected in 57.8% of node-positive patients 3. Breast cancer demonstrates an 8% sentinel lymph node (SLN) positivity rate, with 33.3% of these cases presenting non-SLN metastases 4. LNM is a critical determinant of survival and prognostic outcomes across multiple cancer types, serving as a key indicator of disease progression and therapeutic response. The presence of metastatic tumor cells in regional lymph nodes is strongly correlated with increased recurrence risk, diminished disease-free survival (DFS), and reduced overall survival (OS) 5, 6. In cervical cancer, nodal involvement decreases 5-year survival from 74.8% (T1) to 39.3% (T3) 7. Similarly, renal cell carcinoma with ≥10 positive nodes exhibit a 38% 2-year survival rate compared to 64% for single-node involvement, underscoring the prognostic significance of nodal burden 8. Consequently, positive LNM status typically necessitates aggressive therapeutic interventions.

The treatment of LNM remains a critical challenge in oncology, with current therapeutic approaches increasingly incorporating multimodal strategies that combine surgical, systemic, and localized interventions. However, the limitations of different treatments, their toxic side effects, and the heterogeneity of their efficacy remain critical bottlenecks demanding breakthroughs 9. For instance, external beam radiotherapy (EBRT) is constrained by anatomical limitations and the tolerance thresholds of normal tissues. Minimally invasive interventional therapy, with its unique advantages of precision, efficiency, minimal invasiveness, repeatability, and the ability to overcome hypoxic resistance, has become an essential complementary approach to local treatments. RISB has emerged as a promising minimally invasive brachytherapy modality for managing LNM, particularly in cases resistant to conventional therapies such as surgery, EBRT, and systemic treatments. This technique delivers high-dose radiation directly to the tumor while sparing adjacent critical structures, owing to the rapid dose fall-off of low-energy γ-rays (27-35 keV) emitted by ¹²⁵I seeds 10, 11. Clinical evidence has demonstrated substantial LCR and symptomatic improvement in metastatic lymph nodes across various malignancies, including thyroid, esophageal, and head and neck cancers. For example, in radioactive iodine-refractory differentiated thyroid cancer (RAIR-DTC), ¹²⁵I implantation achieved an LCR of 94.9% at 5 months post-treatment, with concurrent reductions in serum thyroglobulin levels and pain scores (P < 0.001) 12, 13. Similarly, in esophageal cancer patients with recurrent LNM following prior radiotherapy, RISB resulted in a 69.9% tumor response rate and a 2-year local progression-free survival rate of 23% 14, 15. Despite these promising outcomes, several challenges remain, including the suitability of RISB for all LNM cases due to tumor heterogeneity and the necessity for individualized dose optimization 14, 16. For instance, poorly differentiated tumors, such as small cell carcinoma, may require higher initial doses or alternative isotopes like protactinium-103 11, 16. This retrospective study analyzed the efficacy and dose differences of RISB in the treatment of LNM from various cancers with the aim of investigating factors affecting their efficacy and dose optimization. Our study will inform the selection of patients who will undergo RISB for the treatment of LNM and the determination of the appropriate prescribed dose.

## Methods

### Patients

This retrospective cohort study enrolled patients with LNM who underwent RISB at the Sun Yat-sen University Cancer Center from September 2015 to September 2023. The inclusion criteria comprised: (1) age ≥ 18 years; (2) histologically confirmed LNM; (3) treatment with RISB, without external radiotherapy or other local treatments such as ablation before surgery; (4) lesion diameter ≤ 5 cm; (5) efficacy could be accurately evaluated; (6) minimum postoperative follow-up duration of 6 months. Exclusion criteria included: (1) suboptimal seed positioning confirmed by postoperative imaging; and (2) incomplete clinical documentation in medical records.

### RISB procedure

Preoperative assessment involves precise tumor localization through contrast-enhanced computed tomography (CT), with acquired images subsequently integrated into the treatment planning system (TPS). The clinical target volume (CTV) is meticulously delineated, followed by TPS-based calculations of seed quantity and activity required to deliver the prescribed radiation dose. Concurrently, seed spatial distribution is optimized, and organs at risk (OARs) adjacent to the tumor are contoured, thereby finalizing the preoperative treatment plan. Intraoperatively, under CT guidance, seed needles are precisely positioned at predetermined tumor coordinates. Seed implantation is executed using a retraction needle technique with 5-10 mm spacing intervals, strictly adhering to the TPS-generated preoperative plan. Continuous patient monitoring is maintained throughout the procedure, with immediate symptomatic management of any complications. Postoperative evaluation commences with immediate CT imaging for dose verification using TPS analysis. Long-term follow-up incorporates serial imaging studies, with tumor response assessed according to Response Evaluation Criteria in Solid Tumors (RECIST) version 1.1 17. Concurrent monitoring of potential radiation-induced damage to adjacent normal tissues ensures an optimal balance between precise local radiotherapy delivery and patient safety.

### Data

Comprehensive clinical data, postoperative TPS dose verification parameters, and therapeutic efficacy outcomes were systematically collected for all enrolled patients. The clinical dataset encompassed demographic characteristics (age, gender), tumor-related parameters (location, size), surgical details, and histopathological classification of the primary tumor. TPS dose verification metrics included dosimetric parameters such as the prescribed dose received by 90% and 100% of the target area (D90, D100), as well as the target volume covered by 90%, 100%, 150%, and 200% of the prescribed dose lines (V90, V100, V150, V200), complemented by the activity of implanted radioactive seeds. The therapeutic response was evaluated at the 6-month postoperative follow-up, with the ORR calculated as the proportion of complete response (CR) and partial response (PR) cases relative to the total patient cohort, expressed as (CR + PR)/total cases × 100%. Similarly, the LCR was determined by incorporating stable disease (SD) cases, calculated as (CR + PR + SD)/total cases × 100%.

### Statistical analysis

Statistical analyses were performed using R language version 4.3.0 (The R Foundation for Statistical Computing, Vienna, Austria). Continuous variables with normal distribution were presented as mean ± standard deviation and analyzed using independent samples t-test. Non-normally distributed continuous variables were expressed as median (interquartile range) and compared using the Wilcoxon rank-sum test. Categorical variables were reported as frequencies (percentages) and analyzed using either the chi-square test or Fisher's exact test, as appropriate. The optimal cutoff value was determined through receiver operating characteristic (ROC) curve analysis using the Youden index method. Variables were screened using univariate logistic regression analysis (P < 0.05), and these significant variables were subsequently incorporated into multivariate logistic regression analysis to identify independent factors associated with treatment outcomes. All statistical tests were two-tailed, with a p-value < 0.05 considered statistically significant.

## Results

### Baseline characteristics

**Figure 1:**
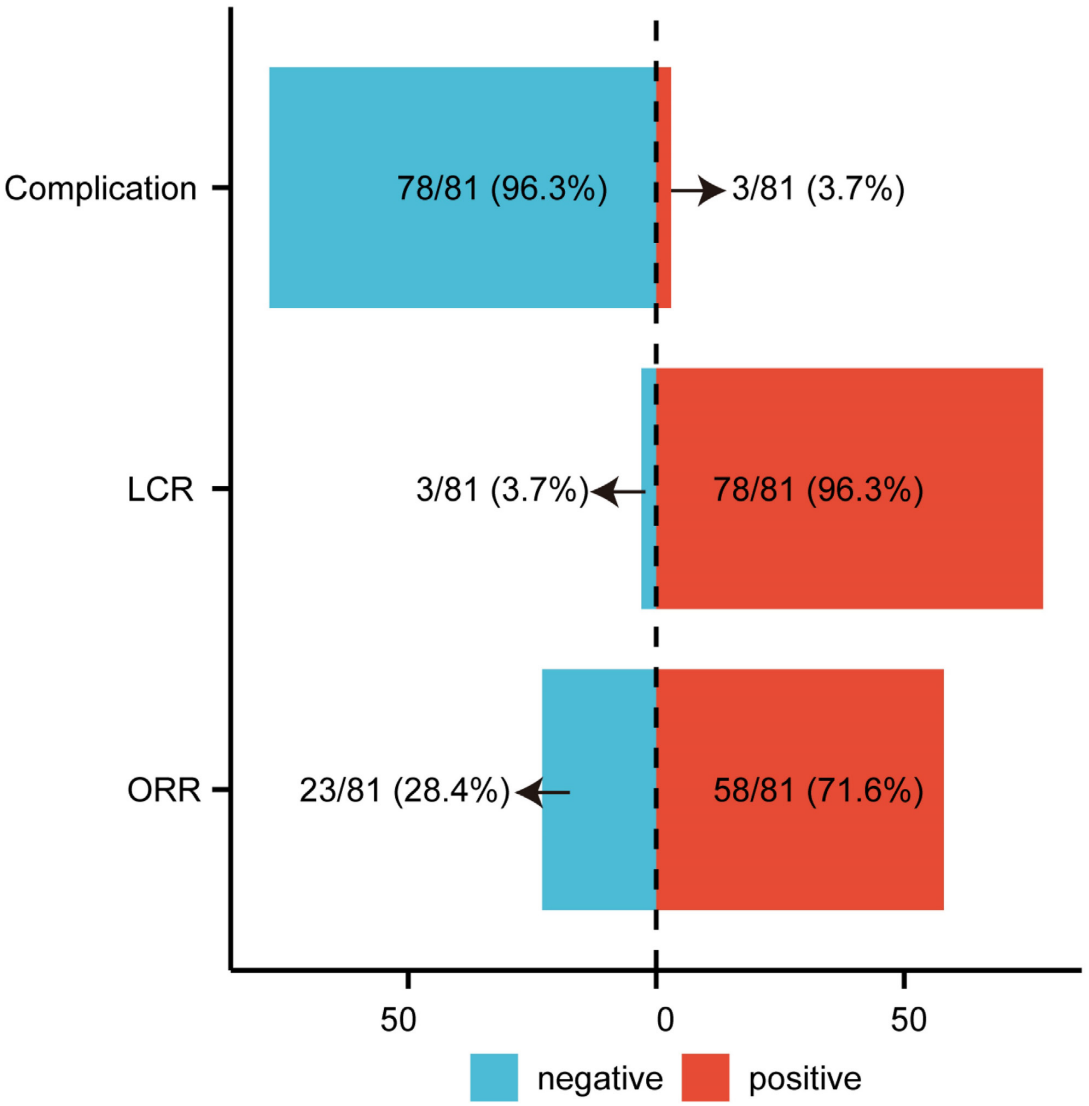
** The Efficacy and Complications of Radioactive Iodine-125 Seed Brachytherapy in the Treatment of LNM.** ORR objective response rate; LCR local control rate; LNM lymph node metastasis.

Following the application of inclusion and exclusion criteria, 81 cases of LNM were enrolled in the study (**Table 1**), with a mean age of 51.49 years. Objective response (CR + PR) was observed in 58 cases (71.60%), while 23 cases (28.40%) exhibited non-objective response (SD + progressive disease [PD]) (**Figure 1**). Baseline characteristics including age, gender, cancer location, and complication rates were comparable between the objective response group and the non-objective response group, but no significant differences were observed (all P > 0.05; **Table 1**). However, significant differences were identified in tumor size, D90, D100, V90, V100, V150, and V200 (P < 0.05). The objective response group demonstrated a smaller median tumor diameter (18.06 mm) compared to the non-objective response group (27.10 mm) (**Table 1**). Postoperative dosimetric parameters (D90, D100, V90, V100, V150, and V200) were significantly higher in the objective response group than in the non-objective response group (**Table 1**). The overall postoperative LCR was 96.3% (**Figure 1**). Complications occurred in 3 cases (3.7%) (**Figure 1**), including one case of minor pneumothorax (**Supplementary Figure 1**) and two cases of minor bleeding. No instances of seed migration, needle tract metastasis, or severe pain requiring pharmacological intervention were reported. The minor pneumothorax resolved spontaneously, and the bleeding was controlled with hemostatic agents. The treatment demonstrated significant efficacy and safety. Pathological analysis identified 18 primary tumor types in LNM cases, with hepatocellular carcinoma being the most prevalent (32.1%) (**Supplementary Table 1**).

### Factors affecting ORR

Univariate and multivariate logistic regression analyses (**Table 2**) demonstrated that tumor size emerged as an independent factor significantly associated with ORR. In contrast, postoperative dosimetric parameters, including D90, D100, V90, V100, V150, and V200, were not identified as independent factors but were observed to exert related influences on ORR. Furthermore, demographic and anatomical variables such as age, gender, and tumor location exhibited no statistically significant correlation with ORR.

### Dose optimization

**Figure 2:**
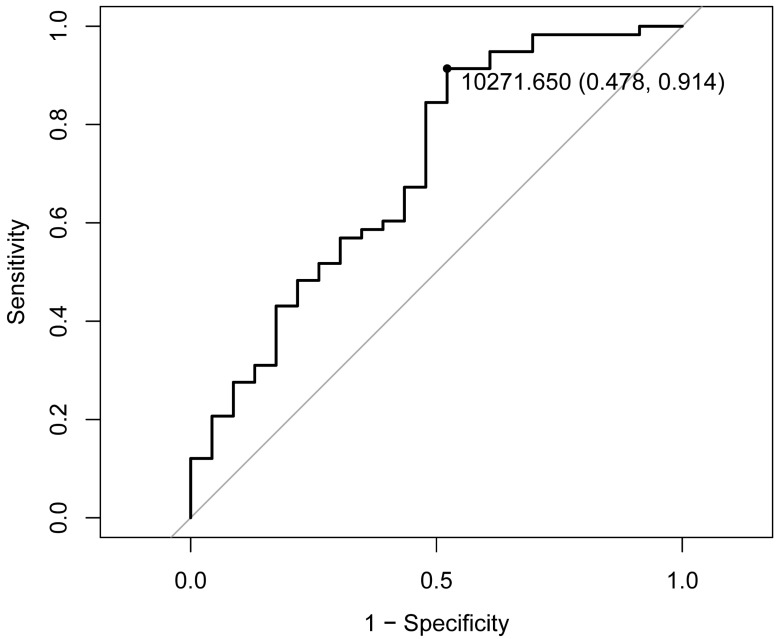
** ROC curve.** ROC analysis identified the optimal D90 threshold as 102.7 Gy. D90 prescribed dose received by 90% of the target area.

The ROC curve analysis, employing the Youden index method, demonstrated that a D90 value of 102.7 Gy represents the optimal threshold for distinguishing the objective response population (**Figure 2**). Based on this threshold, the study cohort was stratified into high-dose (D90 > 102.7 Gy, n = 65, 80.25%) and low-dose (D90 ≤ 102.7 Gy, n = 16, 19.75%) groups (**Table 3**). Statistical analysis revealed a significant difference in ORR between the two groups (P<0.01), with the high-dose group having an ORR of 81.54% and the low-dose group having an ORR of only 31.25% (**Table 3**), which was significantly lower than that of the high-dose group. There were no statistically significant differences in age, gender, tumor location, or complication incidence between the high-dose and low-dose groups (P > 0.05). Notably, while complications were exclusively observed in the high-dose group, statistical evaluation confirmed that a D90 exceeding 102.7 Gy does not significantly increase the risk of complications (P > 0.05). These findings suggest that administering doses above 102.7 Gy in the context of RISB for LNM is both therapeutically effective and clinically safe.

## Discussion

LNM represents a critical event in the progression of numerous malignant neoplasms, manifesting at various stages of tumor development. Beyond its role as an indicator of local tumor dissemination, LNM has been implicated in facilitating distant metastasis through diverse molecular mechanisms 18, thereby significantly influencing patient outcomes 19, 20. RISB has emerged as an effective and safe therapeutic modality for LNM, particularly in cases where surgical intervention is contraindicated, systemic therapies yield suboptimal results or disease recurrence occurs following EBRT. In the present study, RISB demonstrated notable efficacy, with an ORR of 71.6% (44 cases of PR), a LCR of 96.3% (20 cases of SD) at 6 months, and a remarkably low complication rate of 3.7%.

A retrospective analysis of 24 patients with metastatic tumors in the right subclavian tracheal lymph nodes demonstrated an ORR of 87.5% following RISB, including 4 cases of CR and 17 cases of PR, with a significant reduction in the mean diameter of metastatic lymph nodes from 40.21 mm to 12.25 mm (p < 0.001) 21. In a separate study involving 11 patients with iliac LNM, the ORR was 72.73% (8 cases of PR) at the 2-month follow-up, with 2 cases of SD and a LCR of 90.91% 22. Among 15 patients with oral and maxillofacial squamous cell carcinoma who underwent postoperative RISB, the LCR reached 95.3% at 6 months 23. The ORR and LCR observed in our study were generally consistent with those reported in prior investigations 22, 23, although the ORR was lower than that reported by He et al. 21, 24. These discrepancies may be attributed to differences in the types of primary tumors evaluated across studies. Furthermore, our study encompassed a larger sample size and a broader spectrum of primary cancers. Previous research has identified key determinants of treatment efficacy, including radiation dose parameters (e.g., D90), tumor characteristics (e.g., lesion size, primary tumor stage), and short-term treatment response 12, 14. This study corroborates that tumor size is an independent predictor of ORR, while radiation dose parameters, such as D90 and D100, are influential but not independent factors, aligning with earlier findings. We hypothesize that this may be due to an intrinsic relationship between tumor size and achievable dose parameters. As a comprehensive variable encompassing anatomical complexity and biological characteristics, tumor size's potent predictive effect may partially mask or mediate the apparent influence of dose parameters. Chen et al. 25 demonstrated in their study on recurrent retroperitoneal LNM that tumor volumes ≤ 49.8 cm³ were associated with significantly prolonged local control. Collectively, smaller tumor volumes correlate with improved treatment outcomes, likely due to central necrosis in larger tumors resulting from inadequate blood supply, which leads to heterogeneous dose distribution following particle implantation 26, 27.

Previous investigations have not comprehensively addressed the dosage optimization required to enhance therapeutic efficacy, and research in this domain remains limited. Consequently, this study aims to identify the optimal dosage to improve treatment outcomes. Our findings indicate that a postoperative D90 exceeding 102.7 Gy significantly enhances therapeutic efficacy, with the ORR increasing from 31.25% in the low-dose group to 81.54% in the high-dose group. The expert consensus 28 on the prescription dose from the American Brachytherapy Society states that the postoperative D90 should at least reach the prescription dose. Based on these findings, we propose that the prescribed dose for LNM treated with RISB should be at least 102.7 Gy. In recurrent head and neck cancers, a D90 ≥ 130 Gy correlated with superior local control (72.7% for cervical lymph node recurrences vs. 39.9% for primary site recurrences) 29. For thyroid cancer LNM, the recommended prescription dose ranges from 120 to 150 Gy, with no significant difference in D90 between postoperative and preoperative plans 30. Notably, the recommended prescription dose in our study is lower than those reported in prior studies, which may be attributed to the inclusion of diverse tumor types (18 distinct malignancies) in our cohort, as opposed to the recurrent or refractory cases predominantly examined in earlier research.

This study represents a single-center retrospective investigation, with a subsequent multicenter prospective study planned to validate the proposed prescription dosage. Furthermore, although the research encompasses various primary tumor types, the limited sample size for certain tumor categories necessitates the future expansion of the study cohort to enhance statistical power and generalizability.

## Conclusion

This study confirms that CT-guided RISB is an effective and safe local treatment option for LNM. More importantly, through dosimetric analysis, we propose a clear dose optimization target (postoperative D90 > 102.7 Gy) for the LNM population, providing crucial scientific evidence for developing more standardized RISB treatment protocols in the future.

## Supplementary Material

Supplementary figure and table.

## Figures and Tables

**Table 1:** Patient characteristics

Variables	Total (n = 81)	SD^*^+PD (n = 23)	CR+PR (n = 58)	Statistic	*P*
Age (years), Mean ± SD	51.49 ± 11.72	54.52 ± 13.52	50.29 ± 10.83	T = 1.47	0.14
Sex, n (%)				χ² = 0.34	0.56
Male	56 (69.14)	17 (73.91)	39 (67.24)		
Female	25 (30.86)	6 (26.09)	19 (32.76)		
Cancer location, n (%)				-	0.56
head and neck	8 (9.88)	4 (17.39)	4 (6.90)		
chest	11 (13.58)	4 (17.39)	7 (12.07)		
abdomen	53 (65.43)	13 (56.52)	40 (68.97)		
pelvic	5 (6.17)	1 (4.35)	4 (6.90)		
lower limbs	4 (4.94)	1 (4.35)	3 (5.17)		
Tumor size (mm), M (Q₁, Q₃)	19.70 (16.00, 27.80)	27.10 (19.45, 34.90)	18.06 (14.22, 23.02)	Z = -3.47	< 0.01
D90, Mean ± SD	12276.09 ± 2601.80	10807.31 ± 2507.85	12858.53 ± 2420.28	T = -3.40	< 0.01
D100, Mean ± SD	7442.40 ± 2220.36	6334.83 ± 2052.29	7881.61 ± 2145.32	T = -2.96	< 0.01
V90, M (Q₁, Q₃)	0.94 (0.89, 0.97)	0.89 (0.83, 0.95)	0.95 (0.93, 0.98)	Z = -2.71	< 0.01
V100, M (Q₁, Q₃)	0.91 (0.85, 0.94)	0.87 (0.79, 0.92)	0.91 (0.88, 0.95)	Z = -2.72	< 0.01
V150, Mean ± SD	0.68 ± 0.13	0.62 ± 0.15	0.70 ± 0.11	T = -2.92	< 0.01
V200, Mean ± SD	0.51 ± 0.14	0.45 ± 0.15	0.53 ± 0.13	T = -2.41	0.02
Complication, n (%)				-	1.00
without complication	78 (96.30)	22 (95.65)	56 (96.55)		
with complication	3 (3.70)	1 (4.35)	2 (3.45)		

t: t-test, Z: Mann-Whitney test, χ²: Chi-square test, -: Fisher exact, SD: standard deviation, M: Median, Q₁: 1st Quartile, Q₃: 3rd Quartile, CR: complete response, PR: partial response, SD^*^: stable disease, PD: progressive disease, D90/D100: dose received in 90/100 percent of target areas, V90/V100/V150/V200: volume of target area covered by 90%, 100%, 150%, 200% prescription dose lines.

**Table 2:** Univariate and multivariate logistic regression analyses of ORR

Variables	Univariable Analysis			Multivariable Analysis		
	β	*P*	OR (95%CI)	β	*P*	OR (95%CI)
Age	-0.03	0.15	0.97 (0.93 ~ 1.01)			
Sex						
Male			1.00 (Reference)			
Female	0.32	0.56	1.38 (0.47 ~ 4.07)			
Cancer location						
abdomen			1.00 (Reference)			
head and neck	-1.12	0.15	0.33 (0.07 ~ 1.49)			
chest	-0.56	0.42	0.57 (0.14 ~ 2.26)			
pelvic	0.26	0.82	1.30 (0.13 ~ 12.70)			
lower limbs	-0.03	0.98	0.97 (0.09 ~ 10.20)			
Tumor size	-0.13	< 0.01	0.88 (0.82 ~ 0.94)	-0.16	<0.01	0.85 (0.77 ~ 0.93)
D90	0.01	< 0.01	1.01 (1.01 ~ 1.01)	0.00	0.09	1.00 (1.00 ~ 1.00)
D100	0.01	< 0.01	1.01 (1.01 ~ 1.01)	-0.00	0.14	1.00 (1.00 ~ 1.00)
V90	13.28	< 0.01	5.86E+05 (99.55 ~ 3.45E+09)	-24.35	0.48	0.00 (0.00 ~ 7.87E+18)
V100	11.10	< 0.01	6.60E+04 (44.00 ~ 9.89E+07)	35.46	0.35	2.51E+15 (0.00 ~ 9.23E+47)
V150	5.70	< 0.01	298.97 (4.14 ~ 2.16E+04)	-13.27	0.47	0.00 (0.00 ~ 8.71E+09)
V200	4.38	0.02	79.69 (1.84 ~ 3.45E+03)	-2.95	0.78	0.05 (0.00 ~ 4.99E+07)

ORR: objective response rate, OR: Odds Ratio, CI: Confidence Interval, D90/D100: dose received in 90/100 percent of target areas, V90/V100/V150/V200: volume of target area covered by 90%, 100%, 150%, 200% prescription dose lines.

**Table 3:** Characteristics of patients in the high-dose group (D90 ≥ 102.7 Gy) and low-dose group (D90 < 102.7 Gy).

Variables	Total (n = 81)	High-dose (n = 65)	Low-dose (n = 16)	Statistic	*P*
Age (years), Mean ± SD	51.49 ± 11.72	50.57 ± 10.47	55.25 ± 15.70	T = -1.44	0.15
Sex, n (%)				χ² = 0.00	1.00
Male	56 (69.14)	45 (69.23)	11 (68.75)		
Female	25 (30.86)	20 (30.77)	5 (31.25)		
RECIST 1.1, n (%)				χ² = 13.59	< 0.01
PD+SD^*^	23 (28.40)	12 (18.46)	11 (68.75)		
CR+PR	58 (71.60)	53 (81.54)	5 (31.25)		
Complication, n (%)				-	1.00
without complication	78 (96.30)	62 (95.38)	16 (100.00)		
with complication	3 (3.70)	3 (4.62)	0 (0.00)		
Cancer location, n (%)				-	0.98
head and neck	8 (9.88)	6 (9.23)	2 (12.50)		
chest	11 (13.58)	9 (13.85)	2 (12.50)		
abdomen	53 (65.43)	43 (66.15)	10 (62.50)		
pelvic	5 (6.17)	4 (6.15)	1 (6.25)		
lower limbs	4 (4.94)	3 (4.62)	1 (6.25)		
D90, Mean ± SD	12276.09 ± 2601.80	13138.27 ± 2065.63	8773.48 ± 1261.32	T = 8.07	< 0.01

t: t-test, χ²: Chi-square test, -: Fisher exact, SD: standard deviation, CR: complete response, PR: partial response, SD^*^: stable disease, PD: progressive disease, D90: dose received in 90 percent of target areas.

## Data Availability

Due to patient privacy protection, the raw data supporting the findings of this study are available from the corresponding author upon reasonable request.

## Abbreviations

CT: computed tomography
CTV: clinical target volume
CR: complete response
DFS: disease-free survival
D90, D100: prescribed dose received by 90% and 100% of the target area
EBRT: external beam radiotherapy
LNM: lymph node metastasis
LCR: local control rate
NSCLC: non-small cell lung cancer
ORR: objective response rate
OS: overall survival
OARs: organs at risk
PR: partial response
PD: progressive disease
RISB: radioactive iodine-125 (¹²⁵I) seed brachytherapy
RAIR-DTC: radioactive iodine-refractory differentiated thyroid cancer
ROC: receiver operating characteristic
SLN: sentinel lymph node
SD: stable disease
TPS: treatment planning system
V90, V100, V150, V200: target volume covered by 90%, 100%, 150%, and 200% of the prescribed dose lines
