# Supplementary material for: Clinical Outcomes and Individualized Seed Implantation Planning for Iodine-125 Seeds Brachytherapy in Lymph Node Metastases
Source: J Cancer. 2026 Jan 30;17(3):507–14. doi: 10.7150/jca.126692 (PMC13003556; doi:10.7150/jca.126692)
Supplement: Supplementary file 1 — Supplementary figure and table. [file jcav17p0507s1.pdf]

Supplementary Table 1. Cancer characteristics of patients.

Variables	Total (n = 81)	SD+PD (n = 23)	CR+PR (n = 58)	Statistic	*P*
Primary cancer, n(%)				-	0.08
adrenal malignant tumor	2 (2.47)	1 (4.35)	1 (1.72)		
breast cancer	1 (1.23)	1 (4.35)	0 (0.00)		
cervical cancer	7 (8.64)	1 (4.35)	6 (10.34)		
cholangiocarcinoma	12 (14.81)	2 (8.70)	10 (17.24)		
colorectal cancer	7 (8.64)	1 (4.35)	6 (10.34)		
endometrial cancer	3 (3.70)	0 (0.00)	3 (5.17)		
esophageal cancer	2 (2.47)	1 (4.35)	1 (1.72)		
gallbladder cancer	1 (1.23)	0 (0.00)	1 (1.72)		
gastric cancer	1 (1.23)	0 (0.00)	1 (1.72)		
hepatocellular carcinoma	26 (32.10)	8 (34.78)	18 (31.03)		
lung cancer	3 (3.70)	1 (4.35)	2 (3.45)		
melanoma	1 (1.23)	1 (4.35)	0 (0.00)		
nasopharyngeal carcinoma	5 (6.17)	0 (0.00)	5 (8.62)		
ovarian cancer	4 (4.94)	2 (8.70)	2 (3.45)		
pancreatic cancer	1 (1.23)	0 (0.00)	1 (1.72)		
renal cancer	1 (1.23)	1 (4.35)	0 (0.00)		
seminoma	1 (1.23)	0 (0.00)	1 (1.72)		
soft tissue sarcoma	3 (3.70)	3 (13.04)	0 (0.00)		

-: Fisher exact, CR: complete response, PR: partial response, SD: stable disease, PD: progressive disease

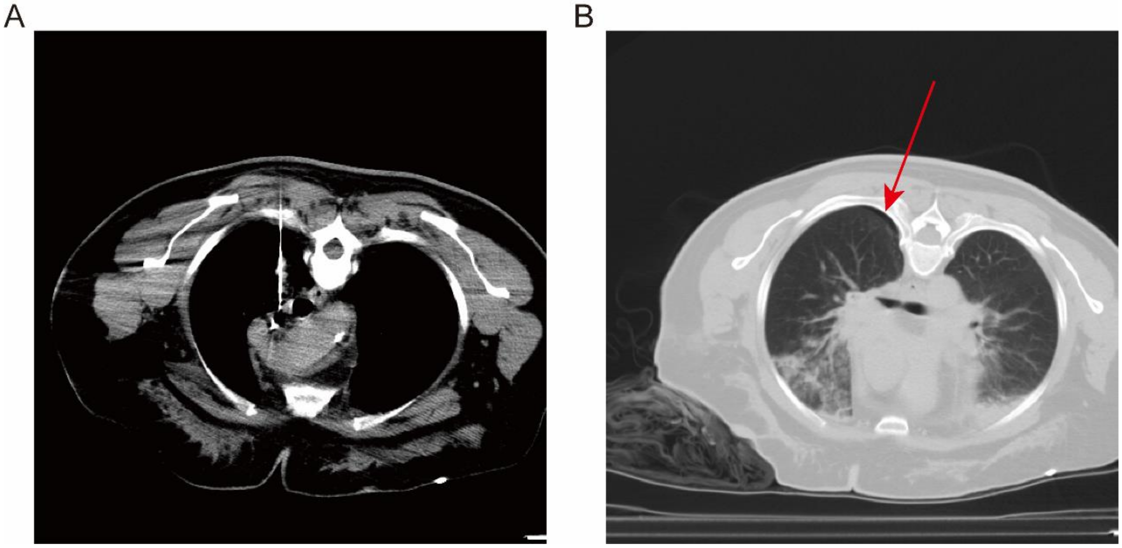

Supplementary Figure 1
